# Molecular players involved in temperature-dependent sex determination and sex differentiation in Teleost fish

**DOI:** 10.1186/1297-9686-46-26

**Published:** 2014-04-15

**Authors:** Zhi-Gang Shen, Han-Ping Wang

**Affiliations:** 1 Aquaculture Genetics and Breeding Laboratory, The Ohio State University South Centers, Piketon, Ohio 45661, USA; 2 College of Fisheries, Huazhong Agricultural University, Wuhan, Hubei 430070, PR China

## Abstract

The molecular mechanisms that underlie sex determination and differentiation are conserved and diversified. In fish species, temperature-dependent sex determination and differentiation seem to be ubiquitous and molecular players involved in these mechanisms may be conserved. Although how the ambient temperature transduces signals to the undifferentiated gonads remains to be elucidated, the genes downstream in the sex differentiation pathway are shared between sex-determining mechanisms. In this paper, we review recent advances on the molecular players that participate in the sex determination and differentiation in fish species, by putting emphasis on temperature-dependent sex determination and differentiation, which include temperature-dependent sex determination and genetic sex determination plus temperature effects. Application of temperature-dependent sex differentiation in farmed fish and the consequences of temperature-induced sex reversal are discussed.

## Background

Sex-determining mechanisms are responsible for a population’s sex ratio, which is the ratio of males to females in the population, a key demographic parameter for its viability and stability. In mammals and birds, embryonic development at the time of sex determination occurs under relatively controlled ambient temperature conditions. In contrast, fish are poikilothermic (cold-blooded) animals and embryonic development takes place in extreme physical environments with relatively marked alternations of temperature. For certain fish species, there is increasing evidence that temperature may affect sex determination [1,2], which could explain the co-evolution of their widespread distribution and wide temperature tolerance range.

In fish, the first evidence of temperature-dependent sex determination (TSD) was presented in 1981 from field and laboratory studies on the Atlantic silverside, *Menidia menidia*[3]. Since then, TSD has been reported for about 60 different fish species belonging to 13 families representative of many types of fishes [4,5]. Different terms are used to describe TSD including “temperature effects on sex ratio”, “temperature effects on sex differentiation”, “temperature influences on sex determination”, “thermolabile sex determination”, “temperature-dependent sex ratios”, “temperature-dependent sex differentiation”, “temperature induced sex reversal”, etc. In species with TSD, there is little information on the genetic differences between sexes. The earliest reported ontogenetic difference between sexes in species with TSD concerns environment-induced fluctuations i.e. changes in ambient temperature during the sensitive (or labile) periods of early development that determine phenotypic sex and sex ratio [4,6]. In vertebrates, genetic sex determination (GSD) and TSD have different temporal patterns i.e. GSD occurs as soon as conception takes place and depends on the genetic constitution of the individual, while TSD occurs later during the thermosensitive period prior to and/or at the beginning of gonadal development [7]. It should be noted that the definition of TSD does not imply that genetic influences on gender are nonexistent because TSD and sex ratio cannot evolve without at least some genetic influence on sex determination [1].

It is not always easy to distinguish sex determination and sex differentiation because in many cases the same criteria based on morphological, cellular, and molecular analyses are used to investigate sex differentiation and to infer the genetic sex of an individual. For the purposes of this review, sex determination is used to describe the genetic and environmental processes and variables that influence sex differentiation, while sex differentiation is used to indicate the physical realization of these events in terms of testicular or ovarian development. The definition of TSD originates from studies on reptiles (lizards and turtles), in which sex differentiation and the thermosensitive period (TSP) occur during the embryogenetic period (incubation) [8-10]. In contrast, in fish species, sex differentiation occurs during the post-embryonic period of larval development (post-hatching). Therefore, the criteria that are used to identify TSD in reptiles cannot be applied in fish. The criteria for distinguishing GSD, TSD and GSD + TE (GSD plus temperature effects) have been extensively discussed by Valenzuela et al. [6] and Ospina-Álvarez and Piferrer [4]. According to the criteria of Ospina-Álvarez and Piferrer [4], only 40 of the 59 fish species for which TSD was claimed on the basis of laboratory and/or field data could be classified as species with TSD, which suggests that TSD is less common than initially thought. In other words, based on their criteria, the sex-determining mechanism of about one third of the fish species that were thought to have TSD may have GSD + TE. Such studies are challenging because they require knowledge on the conditions that are normally encountered in the wild by a particular species or population during the sensitive period of development. Moreover, experimental designs that are aimed at distinguishing between TSD from GSD + TE by mimicking natural temperature fluctuations can be difficult to carry out because extreme conditions may simply alter the process of sex differentiation in a species with GSD only and yield skewed sex ratios [11]. Temperature effects on the sex ratio during the thermosensitive period in a fish species that is claimed to have TSD, should occur as an inheritable trait, i.e. its analysis at the population level should reveal an evolutionary pattern rather than an occasional pattern. However, negative results from studies that test a population for TSD may only reflect the status of a particular geographic population and not the species as a whole, because TSD in fish frequently involves genotype by temperature interaction with strong parental effects on family sex ratio. Indeed, in Atlantic silverside and other fish species, temperature sensitive and insensitive populations that occupy different locations have been observed [1]. In addition, we reported in the bluegill sunfish *Lepomis macrochirus* the existence of two contrasted temperature-sensitive populations (in which the proportion of males increased with either increasing or decreasing temperature) as well as temperature insensitive populations ([12] and personal communication).

As sex-determining mechanisms, TSD and GSD should be considered in an equivalent manner [13], which leads to reconsider the status of fish species that are claimed to have TSD when submitted to extreme temperatures instead of the temperature experienced during development in the wild since changes in sex ratio with temperature variation are ecologically and evolutionally relevant. Furthermore, in zebrafish (*Danio rerio*), it was found that hypoxia can affect sex differentiation and sex development along with down-regulations of various genes that control the synthesis of sex hormones and the increase of the testosterone/estradiol ratio, thus producing male-skewed population versus normoxic groups [14]. It was also shown that hypoxia disrupts primordial germ cell migration during embryonic development through the induction of insulin-like growth factor binding proteins in zebrafish embryos [15,16] and in the Atlantic croaker *Micropogonias undulates* after exposure to natural or laboratory hypoxia [17]. Taken together these results suggest that, in some cases, hypoxia rather than extreme high temperatures may contribute to variation in sex ratio since high temperature is considered to decrease the relative water oxygen solubility and may result in hypoxia [18]. These factors will need to be taken into account in future studies.

**Figure 1:**
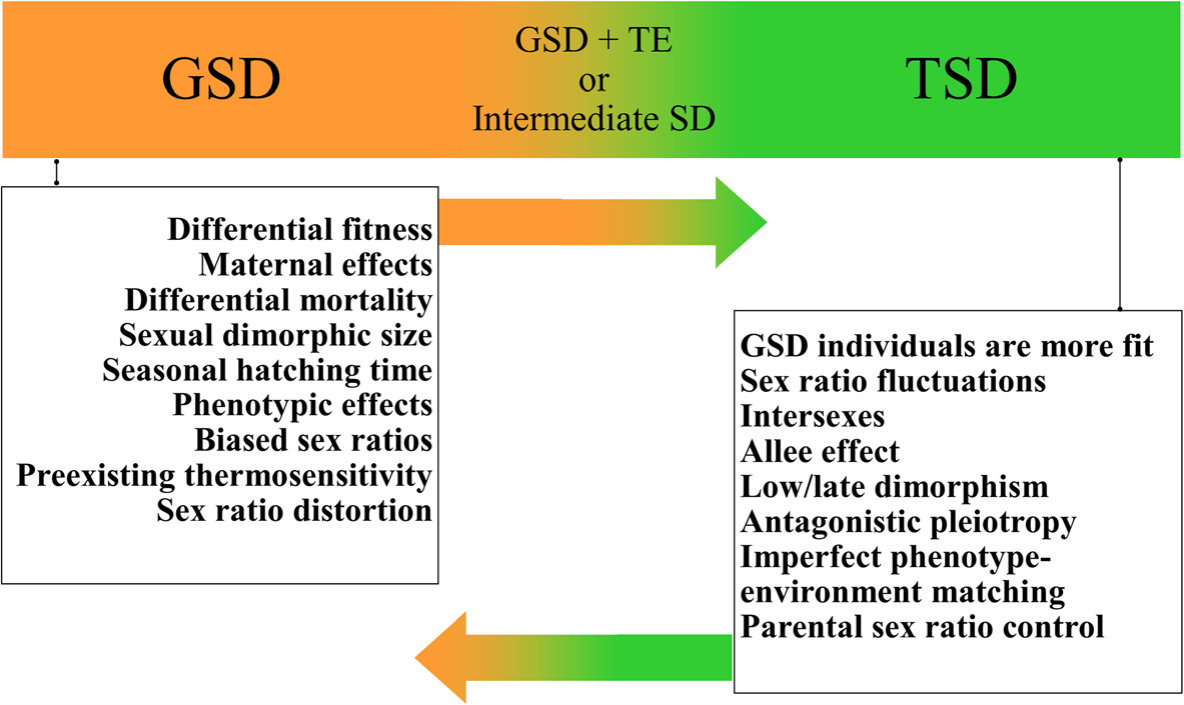
**Transitions between genetic sex determination (GSD) and temperature-dependent sex determination (TSD).** The way the sex-determining (SD) modes are presented does not indicate ancestral states. GSD + TE, genetic sex determination plus temperature effects. The data presented are a compilation from Valenzuela [204].

TSD is said to occur when the water temperature experienced by the offspring irreversibly determines its primary sex [19]. GSD occurs when primary sex is determined by the genotype at conception and is thereafter independent of environmental conditions. How and why transitions between TSD and GSD occur are two key questions about the evolution of TSD. Most of the hypotheses formulated to explain the evolution of TSD are adaptive, but neutral or quasi-neutral alternatives have also been proposed [7] (Figure 1). For example, in the Atlantic silverside, TSD is geography-dependent. In this species, female-biased populations are produced in the early spawning period while male-biased populations are produced later and the change in sex ratio with temperature is greater at low latitudes where the breeding and growing seasons are longer than at high latitudes where the breeding and growing seasons are comparatively short [1]. TSD is adaptive in this fish species because the longer growing season afforded to the female fish allows them to reach a larger size by the time breeding occurs, and a size advantage is more favorable to females than to males [20]. Hence, in Atlantic silverside, both extreme northern and extreme southern populations are GSD (without TSD), while intermediate populations show the maximum level of sex ratio response to temperature (with TSD).

In *Menidia*, it has been shown that TSD is a highly evolved trait that responds rapidly to selection rather than merely the plasticity of a primitive sex-determining mechanism [1]. Schwanz et al. [21] proposed that evolutionary transitions from GSD to TSD (with no sex chromosomes) can occur rapidly and readily through the acquisition of thermosensitivity by selection for TSD without crossing a fitness valley, which was first described by Bull [19,22]. Evolutionary transitions from TSD to GSD are also considered as simple and straightforward [23,24]. Surprisingly, both TSD and GSD have evolved multiple times in the course of vertebrate history via a parsimony-based statistical framework [25]. It was shown that TSD was lost at least six times in turtles and arose at least three times in lizards [25]. Accordingly, since both GSD and TSD have been “lost” and “found” numerous times in vertebrates, the phylogenetic lability of sex-determining mechanisms is extraordinary, which agrees with the above-mentioned hypothesis on transitions between TSD and GSD.

An important aspect to be taken into account is that regardless of whether one species has TSD or GSD (or GSD + TE), under certain circumstances (e.g. under extraordinary natural conditions or when moved to laboratory conditions), fish can respond to temperature changes, which leads to skewed sex ratios. Such effects of the temperature on sex ratios apparently involve the same downstream signaling pathways as TSD. It is possible that, in fish, some steps of the pathways involved in TSD and GSD are similar. Independently of whether sex determination is pure TSD or GSD + TE, it is clear that fish offer an excellent model to explore the plasticity of the mechanisms of sex determination and sex differentiation. Our aim is to standardize the criteria that distinguish TSD and GSD + TE by pooling the data of TSD and GSD + TE together and review the molecular players that underlie the temperature effects in sex determination and differentiation.

## Review

### Putative molecular pathways involved in TSD and GSD + TE

Investigating the genes that are involved in TSD is interesting but information is scarce, even in reptiles. So far, only a few candidate genes associated with temperature-induced sex reversal were studied in fish species. It is assumed that the thermal master switch, which triggers the undifferentiated gonads to follow the male or female pathway, will be the gene(s) that activate the thermosensitive period (TSP) or specify responses during this developmental time window. Candidates for this role would be genes that are expressed prior to, or exactly at, the onset of the TSP, rather than genes that are differentially expressed after TSP activation [26]. Two such potential master switch genes, the *sf-1* and *wt-1* genes that are involved in the formation of a bipotential gonad, have been proposed on the basis of their early significant differential expression before the onset of the TSP in reptiles with TSD. Other genes that have been proposed are *sox9*, *sox8*, *fgf9*, *amh* (*mis*) and *dmrt1* that are associated with the testis-determining pathway, *dax1* and *wnt4* that are involved in intersecting pathways and *foxl2* and *rspo1* that determine the ovarian pathway [27-30]. To our knowledge, none of these genes have been reported to be responsible for TSD or to be direct targets of temperature-dependent sex differentiation in fish. In this article, first we review the genes that are known to play a role in GSD, and then the genes that are expressed downstream in the sex determination cascade and involved in TSD and GSD + TE and in the putative mechanisms that underlie the effect of temperature on sex differentiation. The cortisol-mediated pathway and epigenetic regulatory pathway are also summarized. Our aim is to propose a review of the literature on the master genes involved in TSD to stimulate future investigations.

### Major sex-determining genes involved in GSD

#### The dmy gene

The *dmy* gene is a master sex-determining gene that was first described in 2002 in the medaka, *Oryzias latipes*, which has an XX/XY (female homogamety) sex-determining system [31,32]. It is an excellent candidate as a primary male determining gene equivalent to the *sry* gene of mammals. The gene, designated *dmy*[31] or *dmrt1Y*[32], was located in the Y-specific chromosomal region that contains the male determining gene. It is important to note that this is the only structural gene, which specifies a functional protein in the Y-specific chromosome region [32,33]. The product of this gene contains a DNA-binding domain called the DM domain, a structural motif present in a family of genes that is found in a wide range of invertebrates and vertebrates from nematodes and flies to humans [34-37]. The *dmy*/*dmrt1Y* gene is assumed to have arisen from a recent duplication event (5 to 10 million years ago) of the autosomal *dmrt1* gene [33,38]. A fragment of the medaka linkage group 9 that contains the *dmrt1* gene was duplicated and inserted into the chromosome of linkage group 1, which subsequently became the Y chromosome [39].

The *dmy* gene is expressed before the sex-determining period, specifically, before the first appearance of morphological sex differences at the hatching stage when the male exhibits a decreased number of primordial germ cells [39]. The level of expression of the *dmy* gene during the sex-determining period appears to be critical for its function, since mutants that do not express this gene fail to become males and develop as sex-reversed XY females [31,40]. In sex-reversed XY females induced by estrogen treatment, the *dmy* gene is expressed in the ovaries at a level similar to that in the testes, which means that the expression of this gene is not affected by the administration of exogenous estrogen [32]. Furthermore, the early expression of *dmy* in the somatic cells of the undifferentiated gonad is not influenced by 17β-estradiol (E_2_, natural estrogen) treatment [41]. In addition, a high-temperature treatment (above 27°C) during the sex-determining period fails to induce expression of *dmy* although it leads to the masculinization of medaka XX females [42]. In summary, the expression of *dmy* is in perfect agreement with its function as a male upstream determining gene in medaka.

Two distinct natural mutations in the *dmy* gene present in wild medaka populations have been shown to induce XY genotypes to become fertile females [31]. A subsequent investigation on a natural mutant of medaka fry also showed an increased number of germ cells at day 0 post-hatching due to the low expression of *dmy*, with fry developing into females [40]. A gene knockdown experiment that used gripNA antisense oligonucleotides directed against *dmy* transcripts also showed that *dmy* knockdown XY medaka fry and control XX females had comparable germ cell numbers, which indicates that the disruption of the *dmy* gene resulted in the gonads entering the female pathway [43]. Thus, the *dmy* gene is necessary for the development of males in medaka. Moreover, over-expression of *dmy* by injecting *dmy* genomic DNA fragment into XX female eggs or over-expression of the *dmy* cDNA under the control of the CMV promoter in XX females resulted in XX individuals developing into males [44]. These results indicate that expression of *dmy* may be sufficient to induce male development in XX females.

Collectively, these results suggest that the *dmy* gene is a good candidate male determining gene at least in some of strains of medaka and its relative species *Oryzias curvinotus*[37,40]. In terms of temperature effects on sex determination and differentiation, it is not yet known whether temperature can affect the expression of *dmy*[42] (see next section) although high-temperature treatments during the early stages of development result in females having a male phenotype. It is interesting to note that the thermosensitive period of sex differentiation in medaka lies between developmental stages 5 to 6 (8 to 16 cells) and 36 (heart development stage) [42], which is just before expression of *dmy* begins (stage 36). It is assumed that the function of *dmy* in the male embryo during the sex-determining period is to control the proliferation of primordial germ cells [39]. Likewise, Selim et al. [45] observed that a high-temperature treatment before hatching inhibited the proliferation of germ cells and the development of oocytes and consequently resulted in sex reversal. The fact that *dmy* and temperature have the same effects on the proliferation of primordial germ cells, and that genotypic (XY) males and genotypic (XX) females possess a dimorphic sensitive pattern to temperature during TSP [42] show that temperature may play a pivotal role in female fate as does *dmy* in male fate during evolution. These reports also suggest that genetic as well as environmental factors are not incompatible in terms of effects on sex determination or differentiation with the existence of genotype by environment interactions, as already mentioned in fish species and reptiles [1,4,46,47]. To understand the interaction between the sex-determining gene (*dmy*) and the environment factor (temperature), the effects of temperature on the expression of *dmy* and its downstream targets should be investigated further.

#### The amhy, gsdf, amhr2, and sdY genes

**Figure 2:**
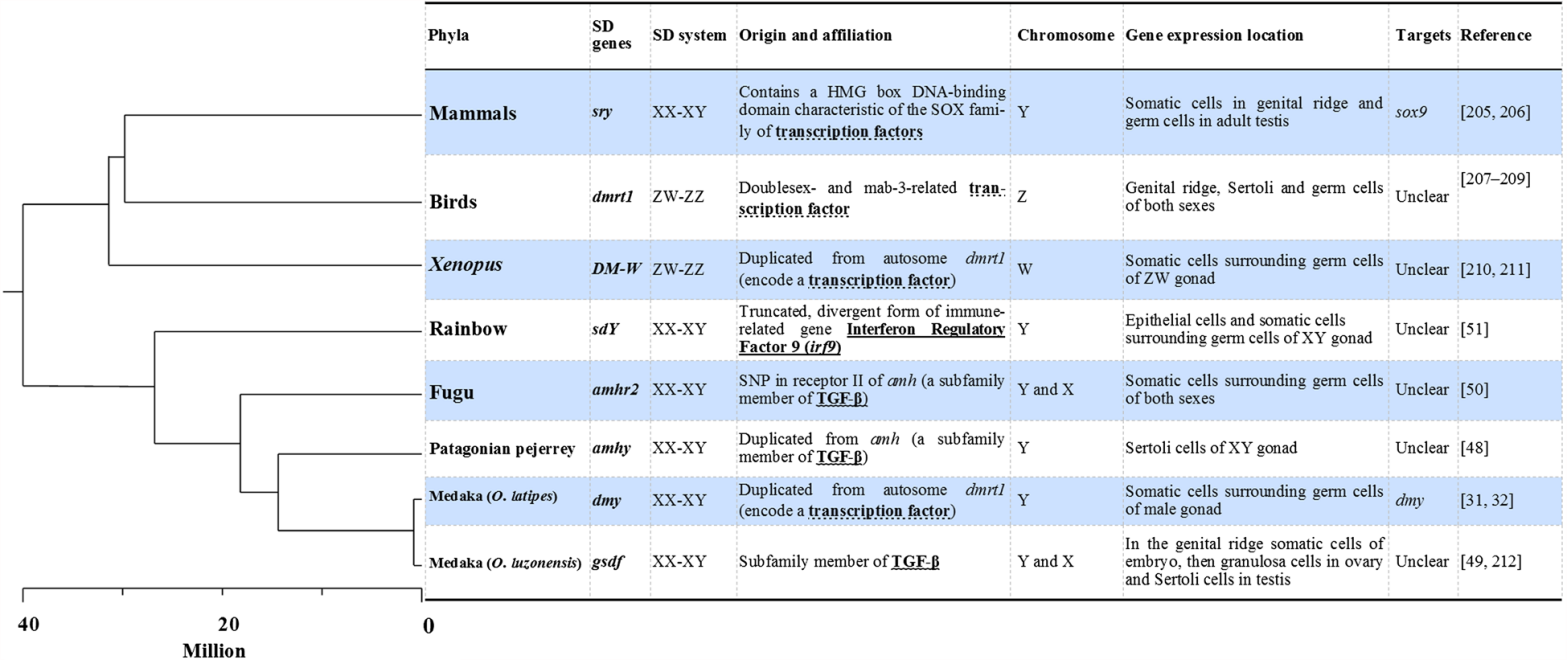
**An overview of master sex-determining genes in mammals, birds and fish.** The phylogeny is a compilation from Kikuchi and Hamaguchi [61]. SD: sex determination; TGF-β, transforming growth factor β; *sox9*: *SRY-like HMG-box containing transcription factor 9* gene; *amh*: *anti-Müllerian hormone* gene (also known as *mis*, *Müllerian-inhibiting substance* gene); *dmy*: *Y-specific DM-domain gene 9* (*dmrt1Y*) [31,32,48-51,213-220].

Just prior to and during the preparation of the present review, four additional strong candidate master sex-determining (SD) genes were reported in fish, which indicates that this area of study is moving fast. These include the genes *amhy* in Patagonian pejerrey *Odontesthes hatchery*[48], *gsdf* in *Oryzias luzonensis*[49], *amhr2* in fugu (tiger pufferfish) *Takifugu rubripes*[50], and *sdY* in rainbow trout *Oncorhynchus mykiss*[51]. All master SD genes reported to date are in Figure 2, which also includes the mammalian *sry* gene, the *dmrt1* gene in birds, the *DM-W* gene in *Xenopus laevis*, and the *dmy* gene in medaka *O. latipes*.

These novel master SD genes highlight the importance of non-transcriptional factors in sex determination since three of these genes i.e. *amhy*, *amhr2* and *gsdf* are involved in the TGF-β signaling pathway, while the *sry*, *dmrt1*, *DM-W* and *dmy* genes code for transcription factors (Figure 2). In mammals, the TGF-β signaling pathway has been shown to play important roles in the development of ovarian and testicular functions [52,53] but there is no evidence that it may be involved in sex reversal. Nevertheless, the identification of the three SD genes *amhy*, *amhr2*, and *gsdf*[48-50] indicates that the TGF-β signaling pathway plays a critical role in the commitment to the fate of either testicular or ovarian development. It has been hypothesized that this pathway may have a more dominant role in gonadal sex determination in non-mammalian vertebrates than in mammals [54]. For example, while *amh* clearly lies far downstream in the sex determination pathway and is controlled by *sox9*, it has been shown in chicken that the expression of *amh* precedes that of *sox9* in males [55,56]. In the American alligator, in which TSD is present, expression of *amh* precedes the onset of *sox9* expression during testis differentiation [57]. Analysis of the expression patterns of *sox9a*, *sox9b* and *sox8* compared to that of *amh* in different cell types ruled out the hypothesis that *amh* is regulated directly by *sox9* or *sox8* at least in the granulosa cells of adult zebrafish ovaries [58]. These findings combined with the aforementioned results in different fish species corroborate or confirm this hypothesis.

To date, one of the four novel SD genes, *sdY*, which is a truncated, divergent form of the immune-related gene *irf9*[51], has not been reported in the literature unlike the three others, which have been characterized and shown to play important conserved roles in the gonadal differentiation pathway across vertebrates. The *sdY* gene, which is expressed in the somatic cells that surround the germ cells, encodes a novel protein that displays sequence homology with the carboxy-terminal domain of *irf9*[51]. IRF9 is a transcription regulatory factor that mediates signaling by type I interferon in mammals [59]. The *sdY* sequence is highly conserved in all salmonids and is a male specific Y-chromosome gene in the majority of these species except in two whitefish species (subfamily *Coregoninae*) [60]. These results indicate that most salmonids share a conserved master sex-determining gene and that an alternative sex-determining system may have also evolved in this family. For more details, refer to Kikuchi and Hamaguchi [61].

Growing evidence demonstrates that the sex-determining pathways in fish species are conserved and diverse (Figure 2, see *O. latipes* and *O. luzonensis*). The *dmy* in *O. latipes* and *gsdf* genes in *O. luzonensis* (a close relative of *O. latipes*) are estimated to have appeared about 10 and 5 million years ago, respectively [49]. It is very interesting to note that the expression of the *gsdf*^*Y*^ gene can lead to a male phenotype in XX medaka *O. latipes* in the absence of *dmy* expression*.* The *gsdf*^*Y*^ gene is located downstream to *dmy* in *O. latipes* and originates from *gsdf*[49], which means that, at present, it functions independently of the existing sex-determining gene, and has usurped the control of the downstream cascade of sex determination within 5 million years.

Genetic by environment interactions are a hot topic. Nevertheless, no evidence has shown that temperature influences expression of sex-determining genes during the TSP. TSD is claimed in more and more fish species but how temperature can act as a signal for the undifferentiated gonad to generate a male or female pathway and what are the downstream target(s) of most sex-determining genes, remain to be elucidated (Figure 2).

### Testis-determining genes involved in TSD and GSD + TE

#### The dmrt1 gene

The mechanisms that control sex determination and sex differentiation are highly variable among different phyla [62]. However, genes that are located downstream in the sex determination pathway are conserved [63]. For example, the *dmrt1* gene plays a pivotal role in the fate of gonads in fish, reptiles, birds and mammals, and is expressed in the developing gonads, or in the adult testis and/or in the ovary. The *dmrt1* gene encodes a putative transcription factor containing a zinc-finger-like DNA-binding motif (DM domain) and was initially identified in nematodes [64] and flies [34]. This gene is regarded as a crucial regulator of male sexual development from invertebrates to humans [65] and also as evidence that the sex determination and gonad differentiation mechanisms are conserved across different lineages [35,66,67].

The ray-finned fish (*Actinopterygii*) has two paralogous copies for many genes (e.g. *dmrt1a* and *dmrt1b*, *cyp19a1a* and *cyp19a1b*, *sox9a* and *sox9b*) due to the hypothesized fish-specific genome duplication that is dated between 335 and 404 million year ago [68]. With the increasing availability of whole-genome sequences, the comparative analysis of genes and genomes will reveal the evolution and phenotypic diversification of the third round (and fourth round in some fish species such as common carp *Cyprinus carpio*) of genome duplication [69-72]. Some duplicated genes have evolved new functions, while others have disappeared [71]. In this review, we focus mainly on the *dmrt1* gene among the many duplicated genes that are mostly related to sex determination and differentiation and have been extensively investigated.

The most direct evidence for the important role of the *dmrt1* gene has come from the discovery that the sex-determining gene of medaka, *dmy* originates from a duplicated copy of the autosomal *dmrt1* gene [44]. In teleost fish, the expression of *dmrt1* is associated with temperature effects and displays variable patterns in different species (See Additional file 1: Table S1). In tilapia (*Oreochromis niloticus*) and trout (*O. mykiss*), *dmrt1* is expressed in males prior to sex differentiation but not in females, which indicates that, in these fish species, it is involved in testis formation and differentiation [73-75]. However, in other fish species, like medaka, pejerrey (*Odontesthes bonariensis*), and European sea bass (*Dicentrarchus labrax*), sexually dimorphic expression of *dmrt1* in males and females was reported (See Additional file 1: Table S1), which indicates that, in these cases, *dmrt1* participates in testis and ovarian development.

Regarding temperature effects on sex ratios, although expression profiles of *dmrt1* have been described in a limited number of fish species, the results suggest that it plays an essential function in male development and testis formation in fish species with TSD or GSD + TE. In pejerrey, a fish species with pure TSD (based on the criteria of Ospina-Álvarez and Piferrer [4]), the abundance of *dmrt1* transcripts differed clearly between larvae reared at female producing (or promoting) temperature (FPT, 100% female) and larvae reared at male producing temperature (MPT, 100% male). The expression of *dmrt1* was significantly higher at MPT than at FPT during the two weeks before the first signs of morphological differentiation of the testis, and remained high during sexual differentiation at MPT, which highlights the importance of *dmrt1* during the first stage of the gonadal sex differentiation cascade, rather than during the morphological differentiation of the gonads. However, it is interesting to note that, in larvae reared at FPT, *dmrt1* expression remained low throughout the experiment (See Additional file 1: Table S1), which opens the question on the function of *dmrt1* in ovarian development in pejerrey [76]. Similar results have been reported in reptiles with TSD [30,77-79].

In red-eared slider turtle (*Trachemys scripta*) embryos, expression of *dmrt1* in the gonads is up-regulated with a large difference between embryos reared at FPT and at MPT, which indicates that *dmrt1* is necessary for male development [80]. In addition, if the eggshells of developing *T. scripta* embryos are treated with estrogen before the thermosensitive period, *dmrt1* expression is inhibited during this period [81]. Suzuki et al. [41] also found that the level of *dmrt1* expression is very low (even undetectable) in testes treated with 17β-estradiol (E_2_, natural estrogen) compared to control untreated testes. In zebrafish, exposure of larvae to environmentally relevant concentrations of 17α-ethinylestradiol (EE_2_, synthetic estrogen) suppresses *dmrt1* expression during gonad differentiation [82]. To our knowledge, there is no evidence on the possible implication of exogenous estrogen treatment on *dmrt1* expression in the induction of sex reversal in fish species with TSD. Taken together, these data strongly indicate that *dmrt1* is involved in testis formation and differentiation, and that its expression is sensitive to both temperature and estrogen in turtles with TSD and fish species with GSD + TE.

Fernandino et al. [83] reported that the expression level of *dmrt1* was not proportional to exposure temperature [83], which suggests that other genes/factors acting as sex inducers and located upstream of *dmrt1* are involved in the transduction of temperature and the gonad differentiation cascade. As in turtles, it is assumed that *dmrt1* still holds the capacity of being a master sex-determining gene in several species with GSD and that it can probably be directly modulated by temperature in species with TSD [30].

Medaka, which is a widely used research model, did not pass the criteria to be diagnosed as a true case of species with TSD because the temperature that causes sex reversal is not within the range of temperatures to which medaka are exposed during the development in the wild [4]. Incubation of medaka at a high temperature (> 30°C) induces sex reversal from genotypic (XX) females to phenotypic males and 100% males are obtained at 34°C [42,84], which means that the sex ratio in medaka is genetically determined with strong temperature effects (GSD + TE). During the temperature-dependent sex reversal of medaka, substantial amounts of *dmrt1* mRNA were detected in six of 12 XX embryos incubated at 32°C but not at the “sexually neutral” temperature of 25°C. Thus, the expression of *dmrt1* could be considered as a marker of sex reversal. This is in a good agreement with the results reported by Hattori et al. [42] who observed 40% of sex-reversed males at 32°C, which confirms that *dmrt1* may have an essential role in temperature-dependent sex reversal from genetic females to phenotypic males. However, other studies on medaka reported that *dmrt1* was expressed at very low levels up to 15-20 days post-hatching (DPH), irrespective of the genetic sex [32,85]. These results lead us to raise two relevant questions: (1) does a high temperature accelerate the cascade of molecular events that lead to testis differentiation (but not ovarian differentiation) and result in earlier expression of *dmrt1*? and (2) is the sex-specific (32°C, embryogenesis) vs. non-sex-specific (possibly 25°C, after hatching) expression of *dmrt1* due to the influence of incubation at high-temperature or is it necessary for natural ovary development? More studies on *dmrt1* expression at various developmental temperatures are necessary to clarify these questions and to understand how this gene contributes to the fate, development, and/or maintenance of the testis as well as the ovary in fish species.

It has been reported that in European sea bass that has GSD + TE, the expression level of *dmrt1* mRNA increased continuously in differentiating and differentiated testes between 150 and 300 DPH, while in adult ovaries it increased until 200 DPH and then decreased to an undetectable level [86], which means that *dmrt1* is not necessary for ovarian maintenance in this fish species. The delayed expression of *dmrt1* suggested that it is neither involved in the formation of the undifferentiated gonad (formed around 90 DPH), nor in the high-temperature induced masculinization mechanism. In contrast, in the Nile tilapia in which the sex ratio is genetically determined with strong temperature effects, *dmrt1* expression presented a rapid up-regulation pattern during the critical period of sex differentiation at high temperature in both the XY and XX male population [5], which implies that in this species *dmrt1* is involved in testicular differentiation and high-temperature induced sex reversal. In the pufferfish (*T. rubripes*), *dmrt1* appears to be involved in the degeneration of germ cells in the ovary, which in turn, causes ovary to testis sex reversal induced by high-temperature exposure during the early development of gonads [87].

**Figure 3:**
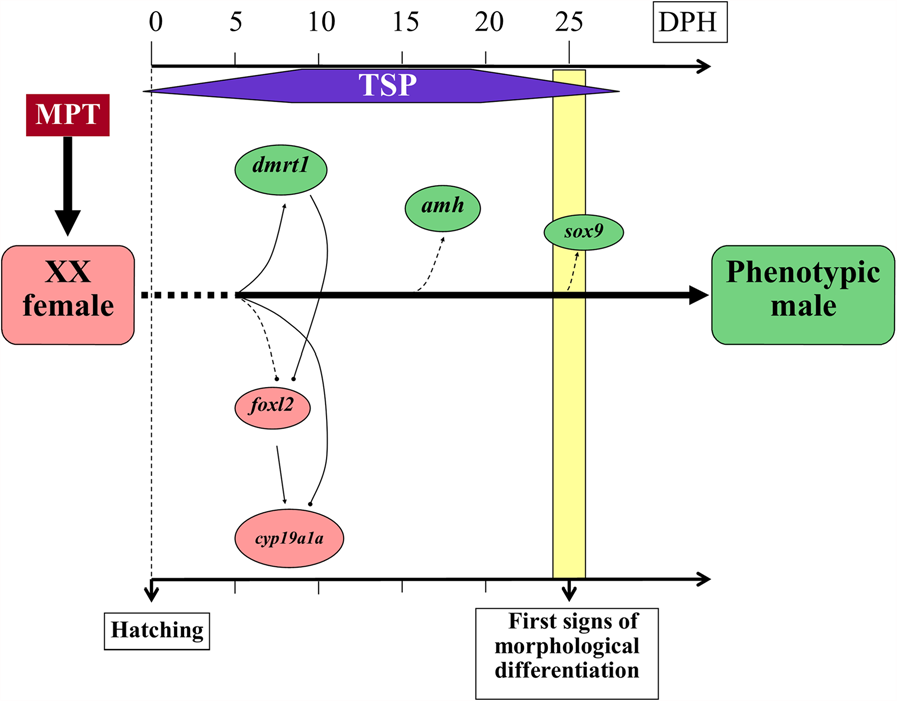
**Putative pathway of temperature-induced sex reversal of XX females towards phenotypic males taking Nile tilapia as an example.** Relationships drawn with solid lines are reported/ demonstrated; dotted lines are putative pathways; arrows indicate up-regulations and lines with dots indicate down-regulations; MPT: male-producing temperature; TSP: thermosensitive period of gonads; DPH: days post-hatching; *dmrt1*: *doublesex- and Mab-3-related transcription factor 1* gene; *amh*: *anti-müllerian hormone* gene; *sox9*: sry-related HMG-box protein 9 gene; *foxl2*: *forkhead transcriptional factor L2* gene; *cyp19a1a*: ovarian *aromatase* gene; data refer to D'Cotta et al. [123], Ijiri et al. [73], Baroiller et al. [5] and Wang et al. [139,144].

Based on the results of the literature on gene expression patterns and on the pathways involved in temperature-induced sex reversal from XX females to phenotypic males in Nile tilapia, we propose a scheme that depicts the putative male pathway induced by high-temperature/MPT (Figure 3). However, how temperature triggers the sex determination of undifferentiated gonads remains to be investigated.

In spite of the diverse and seemingly paradoxical expression patterns of *dmrt1* found among different types of sex determination systems involved in temperature-dependent sex reversal, it is clear that *dmrt1* plays an essential role in testis differentiation and in the temperature signal transduction pathway to the gonad at least in fish species with TSD, and that the up-regulation of *dmrt1* expression is correlated with the temperature-induced male phenotype. Nevertheless, further studies are necessary to elucidate all the mechanisms that involve the *dmrt1* gene.

#### The gene amh/mis

Anti-müllerian hormone (AMH), also known as müllerian-inhibitory substance (MIS), belongs to the transforming growth factor β (TGF-β) family and is secreted by the Sertoli cells. It is responsible for the regression of Müllerian ducts during male fetal development in mammals, birds, and reptiles [88-90] and is involved in both early sex determination and later gonadal development in higher vertebrate species [91]. Although teleost species do not have Müllerian ducts, dimorphic expression of *amh* was detected in developing and/or adult gonads and *amh* seems to play a role in gonadal differentiation and maintenance of both sexes. For example, in Japanese flounder [92], zebrafish [58,93], Atlantic salmon [94] and *Squalius alburnoides* complex [95], *amh* is initially expressed at low levels in the undifferentiated gonads of both sexes and then at higher levels in the testis compared to the ovary during sex differentiation. In medaka, however, this is not the case since from hatching to adult, no sex-specific difference of *amh* expression was found [96].

In reptiles with TSD, *amh* has been studied in the red-eared slider turtle (*T. scripta*) and the American alligator (*Alligator mississippiensis*). In the red-eared slider turtle, expression analyses in both AKG (adrenal-kidney-gonad) complexes and isolated gonads showed that the levels of *amh* expression were higher at MPT than at FTP early in the bipotential gonad and throughout gonadogenesis [80,97]. Moreover, *amh* expression decreased rapidly in gonads shifted from MPT to FPT but not in gonads shifted from FPT to MPT, which suggests that a testis-specific function is repressed and that expression of *amh* is modulated by temperature. In the American alligator, expression of *amh* was only detected in the developing testis of embryos incubated at MPT but not at FPT [57]. The dimorphic expression of *amh* in reptiles suggests that it is involved in temperature-dependent sex determination/differentiation although the mechanism remains unclear.

To our knowledge, the expression level of *amh* has been investigated in only one fish species with TSD, i.e. pejerrey (*O. bonariensis*) [76], in which it is much higher at MPT than at FTP and increases dramatically during gonad differentiation. In addition, a significant increase in *amh* expression is observed in putative males at MPT and at MixPT approximately one week before the first morphological signs of testis differentiation, which is similar to the expression profile in reptiles with TSD. However, the expression level of *amh* was comparable in putative males at MPT and at MixPT and in putative females at FPT and at MixPT, which indicates that, in this fish species, temperature does not modulate directly the expression of *amh* and thus that the expression of *amh* is the consequence rather than the cause of gonad sex differentiation. Thus, other gene(s) or sex inducer(s) are involved in the regulation of gonad sex differentiation in this fish species. Furthermore, it is assumed that *amh* expression is regulated by the level of 17β-estradiol and thus that *cyp19a1* is involved in this regulation [76]. In zebrafish, Schulz et al. [82] reported that exposure to environmentally relevant concentrations of 17β-estradiol during early life suppressed both *amh* and *dmrt1* expression, which was associated with cessation or retardation of testis development.

It is interesting to note that a Y-linked *amh* duplicated copy, termed *amhy* is supposed to be a master male determining gene in the Patagonian pejerrey (*O. hatchery*) [48], which is generally regarded as a species with TSD. The gene *amhy* was located on a single metacentric/submetacentric chromosome in XY individuals and was found to be expressed much earlier than the autosomal form of *amh* (6 days post-fertilization vs. 12 weeks post-fertilization). Furthermore, *amhy* knockdown in XY Patagonian pejerrey embryos results in the up-regulation of *foxl2* and *cyp19a1a* mRNA and the development of ovaries. In the protandrous fish species, black porgy (*Acanthopagrus schlegeli*), expression patterns of *amh* during development and in the adult gonad indicate that *amh* plays important roles in early gonadal development in both sexes and later in ovary growth and natural sex change [98].

The *amh* gene appears to have lost part of its functions and to have acquired new ones, which are involved in sex determination and sex differentiation, during the vertebrate evolution from teleost fish to mammals. It will be interesting to compare the expression profile of *amh* in fish species with TSD and GSD, or fish species with different levels of TSD (varying levels of sex ratio response to temperature, e.g. Atlantic silverside) before and during sex differentiation and in different temperature conditions to address the adaptation of species with TSD. We expect that, in the near future, duplicated copies of other downstream genes involved in sex determination (e.g. *amhy*) will be identified and shown to have essential roles in this mechanism.

#### The sox9 gene

The gene *sox9* is the direct target of the mammalian sex-determining factor SRY [99]. SOX9 and SRY belong to the same family of HMG-box containing transcription factors. SOX9 is a male determining factor both necessary and sufficient for testis formation in mammals. In mice [100,101] and humans [102,103], loss of *sox9* results in male to female sex reversal, while transgenic XX mice that carry a copy of the *sox9* gene develop into phenotypic males [104,105]. Extensively investigated, the function of *sox9* is known to be critical for many aspects of cell differentiation such as heart valve development, neural crest differentiation, chondrocyte specification and male sex determination in mammals [106-109]. Many of these functions have been demonstrated in various vertebrates, which suggests that the *sox9* gene is conserved both structurally and functionally. In several reptiles with TSD, *sox9* was shown to be expressed at higher levels in gonads at MPT than at FPT [110]. The comparison of expression profiles of *sox9* at FPT, MPT, and temperature transfer (FPT to MPT) in reptiles indicates that *sox9* may not be involved in the initial steps of sex determination as in mammals. However, a study on turtles, suggested that its function may be critical for final commitment to a testicular fate [30].

Because of its important and widespread functions, of the ambiguous classification of its orthologs and of the role of its duplication and divergence during the evolution of tetrapod and teleost lineages, most of the studies on the *sox9* gene have focused on these aspects rather than on its role in gonadal determination and differentiation in fish species. The *sox9* gene is present in two copies (known as *sox9a*, *sox9b* or *sox9a1*, *sox9a2*) in most of the fish species studied i.e. medaka, rice field eel, stickleback, zebrafish, fugu and rainbow trout [111-118] as a result of the teleost-specific genome duplication, except in sturgeon (*Acipenseridae*) [119,120]. The expression pattern of *sox9* co-orthologs is more complicated in teleost fish. Without taking into account the ambiguous nomenclature of *sox9* orthologs, the expression patterns and functions of *sox9a* and *sox9b* are likely to be diverse in different fish species in terms of gonadal formation and development (Table 1). Combined with data on reptiles, it seems that *sox9a* or *sox9b* plays a conserved role in gonadal development in non-mammalian species (Table 1). In medaka, during early sex differentiation of the gonads, *sox9b*/*sox9a2* was found to be expressed in the somatic cells surrounding germ cells at comparable levels in both sexes, which suggested that *sox9b*/*sox9a2* is involved in the later development of testis rather than in the early sex determination and differentiation [117]. In addition, a recent highly interesting study that analyzed *sox9b* medaka mutants demonstrated that *sox9b* is not required in testis determination but is indispensable for the proper proliferation and survival of germ cells in both female and male medaka gonads [111]. Another study on air-breathing catfish (*Clarias gariepinus*) showed that *sox9a* was specifically expressed in the developing and adult testis whereas *sox9b* was preferentially expressed in the developing and adult ovary, which confirms that *sox9a* has conserved its function in the testis regarding spermatogenesis while *sox9b* could play a new role in the ovary similar to that in zebrafish (Table 1) [111,114,121,122]. Surprisingly, when catfish individuals were treated with an androgen (11-KT), the level of *sox9a* transcripts increased significantly in adult testicular slices but the mechanism remains to be elucidated [121]. Another interesting report in rice field eel (*Monopterus albus*), which undergoes natural sex reversal from females to males, suggested that the double dose of the *sox9a* genes (*sox9a1* and *sox9a2*) may play a role in gonadal differentiation from female to intersex to testis during sex reversal [112]. In Nile tilapia [73], it was shown that the testis-specific expression of *sox9* only occurred in the later stages of testis differentiation, which supports the hypothesis that *sox9* is involved in testis formation rather than in male determination or differentiation. Another study also on Nile tilapia [123] reported that *sox9b* was strongly expressed in the high-temperature-treated females as early as 12 days post-fertilization (before the first signs of morphological differentiation, Figure 3) and was up-regulated thereafter, which indicates that *sox9b* participates in the temperature-dependent masculinization process. Future work should be aimed at investigating the function of *sox9* in sex commitment in fish species and its divergence during the vertebrate evolution from teleost fish to mammals.

**Table 1:** **Gonadal *sox9 *** **expression in fish**

**Species**	**Co-orthologs**	**Developing testis**	**Developing ovary**	**Adult testis**	**Adult ovary**	**Reference**
**Medaka**	*sox9a*	-	+++	-	+++	[115]
*Oryzias latipes*	*sox9a2*	+++	+++	+++	-	[117]
		↔	↓			
	*sox9a*	NR	NR	+	+++	[122]
	*sox9b*	NR	NR	+++	-	[122]
	*sox9b/sox9a2*	+	+	NR	NR	[206]
**Zebrafish**	*sox9a*	NR	NR	+++	-	[114]
*Danio rerio*	*sox9b*	NR	NR	-	+++	[114]
	*sox9a*	+	NR	+	NR	[58]
	*sox9b*	NR	+	NR	+	[58]
	*sox9a*	NR	NR	++	-	[122]
	*sox9b*	NR	NR	-	+++	[122]
	*sox9a*	NR	NR	+	+	[111]
	*sox9b*	NR	NR	-	+	[111]
**Rainbow trout**	*sox9*	NR	NR	+++	-	[118]
*Oncorhynchus mykiss*	*sox9a1*	+++	+	NR	NR	[74]
		↓	↔			
	*sox9a2*	+++	++	NR	NR	[74]
		↔	↓			
**Siberian sturgeon**	*sox9*	+	+	NR	NR	[120]
*Acipenser baerii*						
**Amur sturgeon**	*sox9*	+	+	NR	NR	[207]
*Acipenser schrenckii*						
**Common carp**	*sox9b*	NR	NR	+++	+	[208]
*Cyprinus carpio*						
**Nile tilapia**	*sox9a*	++	+	+	-	[217]
*Oreochromis niloticus*						
**Yellow catfish**	*sox9a1*	NR	NR	+	+	[209]
*Pelteobagrus fulvidraco*	*sox9a2*	NR	NR	-	+	
**Airbreathing catfish**	*sox9a*	+++	-	+++	-	[110]
*Clarias gariepinus*	*sox9b*	-	+++	-	+++	
**Orange-spotted grouper**	*sox9*	NR	NR	+	+	[211]
*Epinephelus coioides*						
**Rice field eel**	*sox9a1*	NR	NR	+++	+++	[112]
*Monopterus albus*	*sox9a2*	NR	NR	+++	++	

NR: not reported. -: not expressed; +: expressed; the number of + shows the expression levels compared with other studies from the same authors (self-comparison). ↓ and ↔ indicate decrease and invariant of expression level, respectively.

### Ovary-determining genes involved in TSD and GSD + TE

#### The cyp19a1a gene

Due to the important role of estrogen in development, growth and reproduction in teleost fish, aromatase, which is the key enzyme that catalyzes the formation of estrogen from androgen, has been extensively analyzed in the undifferentiated, differentiating, and differentiated gonads as well as in adult fish [124,125]. The gene that encodes aromatase is a duplicated gene in all investigated teleost fish [126-129], except in the eel which belongs to the ancient group of Elopomorpha [130]. The gene duplication gave rise to two different genes (isoforms), namely *cyp19a1a* and *cyp19a1b*, in most teleost fish. The *cyp19a1a* gene is also known as “*gonadal aromatase*” or “*ovarian aromatase*” (also referred to as *p450aromA*, *cyp19a* or *cyp19a1*) since it is mainly expressed in the differentiating and adult gonad of teleost fish. The *cyp19a1b* gene is called the “*neural aromatase*” or “*brain aromatase*” (also referred as *P450aromB*, *cyp19b* or *cyp19a2*) since it is highly expressed in the brain of both male and female teleost species [131] but no sexually dimorphic brain expression during gonad sex differentiation has been demonstrated [132]. Our review of the literature is restricted to studies on the ovarian gene (*cyp19a1a*) with regard to sex differentiation.

Expression of *cyp19a1a* has been detected prior to sex differentiation in all fish species investigated and is associated with temperature-induced sex reversal (See Additional file 1: Table S1). Kitano et al. [133] have studied the Japanese flounder (*Paralichthys olivaceus*), in which sex is genetically determined with strong temperature effects since high temperatures induced an all-male population from an all-female population. They reported that, in this species, the expression of *cyp19a1a* mRNA was the same between males and females during the sex-undifferentiated period up to 50 DPH but that 10 days later when gonads start to differentiate, a specific expression was detected in the females. The level of *cyp19a1a* expression increased rapidly in the female group but decreased slowly in the male group. A subsequent study conducted by the same team on Japanese flounder showed that follicle-stimulating hormone (FSH) signaling and FOXL2 are involved in the transcriptional regulation of the *cyp19a1* gene during gonad sex differentiation [134]. These results indicate that *cyp19a1a* is involved in temperature-induced sex reversal and plays an essential role in ovarian differentiation. However, it seems that *cyp19a1a* is a downstream gene in the sex differentiation pathway and thus, other upstream genes probably trigger the development of the undifferentiated gonads to ovaries or testes in natural sex differentiation or temperature-induced sex differentiation.

In Nile tilapia, in which high temperature causes 100% masculinization, the difference in *cyp19a1a* expression between sexes (higher in females than in males) was shown to exist before the histological differentiation of the ovary and to occur when germ cells enter meiosis [135]. Furthermore, the expression of *cyp19a1a* followed an interesting pattern: higher in all-female (27°C) than in all-female (35°C) than in all-male (27°C) than in all-male (35°C), which indicates that *cyp19a1a* is involved in the high-temperature induced masculinization (See Additional file 1: Table S1). Similar results were also observed in Atlantic Halibut (*Hippoglossus hippoglossus*) in which expression levels of *cyp19a1a* were lower before sex differentiation (13°C < 10°C < 7°C) in the high-temperature treatment group [136]. In addition, the expression of *cyp19a1a* increased in the low-temperature group (7°C) compared to the higher-temperature groups. However, in European sea bass (*Dicentrarchus labrax*), no significant difference in the expression of *cyp19a1a* was detected between MPT and FPT prior to and during sex differentiation (See Additional file 1: Table S1), although the proportion of males was 73% in the high-temperature group compared to 23% in the low-temperature groups [137,138]. In Nile tilapia, *foxl2* was found to strongly activate the transcription of *cyp19a1a* by binding to the sequence ACAAATA in the promoter region of the *cyp19a1a* gene directly through its forkhead domain in *vivo* and in *vitro* studies [139]. In a subsequent study by Navarro-Martín et al. [138] in the European sea bass, methylation of the *cyp19a1a* promoter activated by high-temperature resulted in a lower expression of *cyp19a1a* in temperature-masculinized fish, by preventing binding of *foxl2* and *sf-1* to their sites, and in turn, blocking transcriptional activation of *cyp19a1a*, which agreed with previous studies on *foxl2* and *sf-1*[139-143]. Interestingly, in Nile tilapia, the suppressive effect of *dmrt1* on *cyp19a1a* expression was confirmed *in vivo* and *in vitro* via the repression of the activity of Ad4BP/*sf-1*, which suggests that *dmrt1* suppressed the female pathway by repressing the transcription of the *aromatase* gene and production of estrogen in the gonads [144].

In summary, in temperature-dependent sex determination of phenotypic males, temperature-induced methylation of the *cyp19a1a* promoter or temperature-induced high expression of *dmrt1* are the cause of the down-regulation of *cyp19a1a* expression and the subsequent low level of estrogen. However, the status and relationship between *dmrt1* and methylation of the *cyp19a1a* promoter are unclear and *cyp19a1a* is probably not the direct target of temperature. To our knowledge, in fish species, expression of *cyp19a1a* is generally thought to respond to temperature, namely, it is repressed with increasing temperatures (See Additional file 1: Table S1), which agrees well with the single sex ratio pattern dependent on temperature proposed by Ospina-Álvarez and Piferrer [4]. It could be argued that *cyp19a1a* suppression is the consequence rather than the cause of the suppression of female development. Nevertheless, *cyp19a1a* could be a good “indicator” to differentiate females from males prior to and/or during sex differentiation in some fish species. Undoubtedly, *cyp19a1a* plays an important role in sex differentiation in fish species.

#### The foxl2 gene

FOXL2 is a forkhead domain transcription factor, which is required for granulosa cell differentiation and ovarian maintenance [145]. Mutations of the *foxl2* gene are responsible for blepharophimosis-ptosis-epicanthus inversus syndrome characterized by a distinctive eyelid abnormality and premature ovarian failure [146]. During the past decade, many studies have investigated the expression profiles of *foxl2*, its targets and its signaling pathway from mammals to teleosts and confirmed its pivotal role in the development and maintenance of female sexual characteristics.

Human FOXL2 and SF-1 (SF-1 is a steroidogenic factor-1, also known as Ad4BP and officially designated NR5A1) proteins interact in ovarian granulosa cells i.e. FOXL2 down-regulates the transcriptional activation of the steroidogenic enzyme, CYP17, through SF-1 [147]. Furthermore, patients with blepharophimosis-ptosis-epicanthus inversus syndrome type I present mutations in the *foxl2* gene that result in the loss of the ability to suppress the induction of *cyp17* mediated by SF-1. This demonstrates that mutations in the *foxl2* gene are responsible for the disruption of normal ovarian follicle development. In goats, FOXL2 has been shown to be a direct transcriptional activator of the *cyp19* gene via its ovarian-specific promoter 2 [148]. Goats with polled intersex syndrome in which the function of *foxl2* is disrupted have a reduced expression of aromatase compared to control animals [149,150]. The phenotype of *foxl2* knockout mice comprises total absence of secondary follicles and oocyte atresia [145,151] and mouse XX gonads without *foxl2* develop into males [152], which suggests that *foxl2* represses the male pathway during female gonadal development. For more details on the *foxl2* function in ovarian development in mammals, refer to the review by Uhlenhaut and Treier [153].

To date, in fish species, analyses of the expression of *foxl2* reveal a sex-specific pattern and a positive correlation with the expression of *cyp19a* (Table 2). In particular, it has been reported in Nile tilapia, that *foxl2* can directly activate the transcription of *cyp19a1* and also interact with *sf-1* to strengthen the *sf-1* mediated *cyp19a1* transcription [139]. Moreover, disruption of the endogenous *foxl2* gene in XX tilapia individuals induces occasional sex reversal from ovary to testis with a down-regulated expression of *cyp19a1* and reduced serum levels of 17β-estradiol. In Japanese flounder, it is hypothesized that *foxl2* may directly induce the expression of *cyp19a*[134]. On the contrary, in medaka in which there is a significant delay between the onsets of the expressions of *foxl2* and *aromatase*, expression of *foxl2* alone may not be sufficient to induce expression of *aromatase* and other factors might be involved [154]. In rainbow trout [155] and southern catfish [156], treatment with 17β-estradiol up-regulates the expression of *foxl2* while exposure to an aromatase inhibitor suppresses it, which suggests that the expression of *foxl2* is regulated through a feedback mechanism of downstream hormones. Taken together these results indicate that *foxl2* plays a key role in female gonadal differentiation and maintenance through the activation of *cyp19a* and subsequent estrogen synthesis.

**Table 2:** ***foxl2 *** **expression profile and putative signaling pathway**

**Species**	***foxl2 *** **sex-specific expression during sex differentiation**	**Positively correlated with *cyp19a***	**Positively correlated with *sf-1***	**Regulate *cyp19a *** **directly**	**Regulate *cyp19a *** **by *sf-1***	**Thermosensitive**	**Reference**
Nile tilapia	**√**	**√**	**×**	NS	NS	NS	[73]
Japanese flounder	**√**	**√**	NS	**√**	NS	**√**	[134]
Nile tilapia	**√**	**√**	**√**	**√**	**√**	NS	[139]
Medaka	**√**	NS	NS	NS	NS	NS	[154]
Airbreathing catfish	**√**	**√**	NS	NS	NS	NS	[212]
Rainbow trout	**√**	**√**	NS	NS	NS	NS	[155]

√:expressed; **×**:not expressed; NS: not studied.

In Japanese flounder, in which there is complete sex-reversal from males to females or from females to males by exposure to high (27°C) or low (18°C) temperatures during TSP respectively [133], expression of *foxl2* in the gonads is suppressed at high temperature [134]. This indicates that *foxl2* may also act as a female determinant in fish species with TSD.

### Other pathways

Pathways other than the classical sex differentiation pathways have been reported to be associated with sex differentiation in fish species with TSD or GSD + TE during the past decades. The heat shock protein (HSP70) family includes multiple members, which may reflect the evolutionary potential of fish species for adapting to changes in the environment and in particular to physiological modifications linked with sex reversal. In the swamp eel (*Monopterus albus*), the expression pattern of one member of the HSP70 family, HSPA8B2, mainly detected in the testis supports the gene *hspa8b2* as a candidate in gonadal development/spermatogenesis [157]. Rissanen et al. [158] reported that, in crucian carp, hypoxia-inducible transcription factor-1, a master regulator of hypoxia-induced gene responses, was involved in the control of the gene’s responses to both oxygen and temperature, which may indicate that it participates in sex differentiation since hypoxia disrupts primordial germ cell migration and influences sex differentiation. Because the amount of information on these novel pathways is quite limited, we focused our review on the cortisol-mediated pathway and the epigenetic regulatory pathway.

#### The cortisol-mediated pathway

Hormones are considered as the primary communicators between external conditions and physiological activities because environmental information must first be transduced into a physiological signal to influence sex ratio [159]. As early as 1985, van den Hurk and van Oordt found [160] that, in rainbow trout larvae, exposure to cortisol and cortisone inhibited ovarian growth and increased the proportion of males. Cortisol is the major glucocorticoid produced by the interrenal cells and is used as an important indicator of stress since its production is increased by stressors such as handling, acid water and rapid temperature changes in fish [161]. Cortisol regulates a diverse array of systems including metabolism, ion regulation, growth and reproduction [162].

In recent years, several studies reported that exposure to high temperature elevated cortisol levels and led to the masculinization of fish species with TSD and GSD + TE. In 2010, Hayashi et al. [163] reported that, in medaka, exposure to a high-temperature (33°C) induced masculinization of XX females by elevating the cortisol level, which, in turn, suppressed germ cell proliferation and expression of *fshr* mRNA. Thus, cortisol can cause female-to-male sex reversal in this species. In Pejerrey, a fish species with TSD, individuals treated with cortisol presented elevated levels of 11-ketotestosterone (11-KT) and testosterone and typical molecular signatures of masculinization including up-regulation of *amh* expression and down-regulation of *cyp19a1a* expression [164]. Moreover, in the same species, it was observed that during high-temperature-induced masculinization, cortisol promoted the production of 11-KT by modulating the expression of *hsd11b2*. Cortisol also produces a dose-dependent sex reversal from females to males in the southern flounder (*Paralichthys lethostigma*) i.e. exposure to high (28°C) and low (18°C) temperatures produced a preponderance of males while an intermediate temperature (23°C) favored a 1:1 sex ratio [165]. In addition, in the Japanese flounder, exposure to cortisol caused masculinization by directly suppressing the expression of *cyp19a1a* mRNA by disrupting cAMP-mediated activation [166].

These results provide evidence on the relationships between temperature conditions and the responses of the organism and allow us to draw a picture of the endocrine-stress axis in terms of gonadal fate under temperature effects. They suggest that cortisol may be the “lost” link between temperature and the sex-determining mechanism in species with TSD and may, as a stressor indicator, be involved in the adaptive modification of sex ratio in a spatially and temporally variable environment during the evolution of such species. The relation between glucocorticoid production and androgen production during the masculinization process should be further investigated.

#### The epigenetic regulatory pathway

Epigenetics, a “hot spot” area of biology, is the focus of many studies. Epigenetics is defined as “the study of mitotically and/or meiotically heritable changes in gene function that cannot be explained by changes in DNA sequence” [167]. Epigenetic mechanisms that are involved in the regulation of gene expression typically include DNA methylation, which is relatively well-documented, modification of histones and histone variants, and presence of non-coding RNAs [168].

Gorelick [169] predicted that different methylation patterns of virtually identical sex chromosomes in species with TSD could be the result of small environmental changes (e.g. temperature variation), thereby determining the sex of each individual. He also hypothesized that homomorphic sex chromosomes are required to explain the origin of TSD; sex differences are initially determined by the different methylation patterns of nuclear DNA in females and males, which result in different sexual phenotypes of TSD. Based on an analysis on European sea bass, a fish with a polygenic system of sex determination in which genetics and temperature contribute almost equally to the sexual phenotype [170,171], Navarro-Martín et al. [138] reported that the methylation levels of the promoter of the gonadal *cyp19a1* gene are sex-specific and influenced by the temperature at which one-year-old juveniles are reared. The inverse relationship between methylation levels and *cyp19a1* expression indicates that temperature-induced masculinization (high-temperature) involves DNA methylation-mediated control of *cyp19a1*. These findings are the first example of an epigenetic mechanism that regulates temperature effects on sex ratios in a vertebrate. Recently, similar but slightly different results were also found in red-eared slider turtle, a species with clear TSD [172]. DNA methylation levels of the promoter region of the *cyp19a1* gene were significantly higher in embryonic gonads at the male-producing temperature (26°C) than at the female-producing temperature (31°C). Nevertheless, a switch from male-to-female rather than from female-to-male producing temperature during TSP significantly decreased the level of DNA methylation of the *cyp19a* promoter region in the gonads. These results indicate that temperature, specifically female-producing temperature, causes demethylation of the *cyp19a* promoter region which, in turn, leads to the temperature-specific expression of *cyp19a* and favors the female pathway. Moreover, in the protandrous fish species, black porgy (*Acanthopagrus schlegeli*), it has been reported that the methylation level of the *cyp19a1* promoter was higher in inactive ovaries than in active ones, which suggests that the expression of *cyp19a1* is controlled by an epigenetic mechanism in addition to the classical transcriptional activators of *cyp19a1* such as SF-1 and FOXL2 [173].

Based on these studies, it is tempting to speculate that methylation of the *cyp19a* promoter may participate in the mechanism that links environmental temperature and sex ratios in vertebrate species with TSD but future research is necessary to support this hypothesis. In 2013, Piferrer published an extensive review on the epigenetic regulation of sex determination and gonadogenesis [174]. It would be very interesting to investigate whether the methylation patterns of related genes are conserved in vertebrates with different sex-determining mechanisms including GSD, TSD, as well as hermaphrodite species. We believe that the epigenetic regulation of sex determination and differentiation should be extensively studied in many vertebrates and that such studies would provide new insights on our present understanding of the origin, evolution, and maintenance of sex-determining mechanisms.

### Future studies

During the past two decades, temperature effects on sexual fate have received much attention. Temperature-dependent sex differentiation in fish species with GSD + TE, which is considered as environmental sex reversal (ESR), has been well evaluated in terms of population dynamics but the effects in the natural environment are poorly understood. Temperature-dependent sex differentiation (using Trojan sex chromosome, TSC) is used to produce mono-sex populations in aquaculture [11,175], to selectively breed one sex by increasing the proportion of individuals with the desired sex via exposure to a given temperature [176,177] and to promote the conservation of small and endangered populations, and the biological control of invasive/introduced species (see reference below). The genetic risk associated with stock enhancement of fish with TSD, for example in the Japanese flounder, [178], or the consequences of ESR induced by climate changes (temperature variation) on populations [179], have been theoretically evaluated. Here, we examine the possibility of applying selective breeding to increase the proportion of individuals with the desired sex via temperature treatment, summarize briefly the consequences of the existence of the so-called Trojan sex chromosome in terms of fish population dynamics (specifically the farmed fish species), and propose our ideas on the adaptation of fish populations to changes in global temperature.

Temperature is considered as a consumer- and environment-friendly instrument with applications in selective fish breeding. The first experimental evidence that variation in sex ratio response to temperature can be used in selection designs was obtained in *Menidia*[180,181]. Then, parental, strain, and/or population influences (genotype-temperature interactions) of temperature effects on sex ratio were confirmed in several fish species with sexual dimorphic growth patterns, e.g., bluegill sunfish [12], Nile tilapia [176,182-184], rainbow trout [177], European sea bass [185] and turbot (*Scophthalmus maximus*) [186]. The proportion of females or males after applying a temperature treatment could be significantly increased by selecting the individuals that responded best in Nile tilapia [176,182-184] and rainbow trout [177]. These results support the application of this consumer- and environment-friendly method i.e. temperature treatment, in selective breeding of mono-sex populations or populations with a high proportion of individuals with the desired sex. Although the gene(s) responsible for TSD have not been identified, TSD is proven to be a highly evolved trait that responds rapidly to selection rather than a plasticity of the primitive sex-determining mechanism [1]. The availability and reasonable cost of next-generation sequencing will facilitate the detection of gene(s) responsible for TSD, as well as the identification of sex ratio thermosensitive populations.

In Japanese flounder, which has a XX/XY sex-determining system, sex differentiation is greatly influenced by rearing temperature during the thermosensitive period [133]. High (27°C) or low (18°C) temperatures can produce all-male or all-female populations, respectively. Considering the wide range of rearing temperatures used for Japanese flounder in experimental conditions (12-28°C) [133] or in farm conditions (up to 32°C) [187], ESR probably occurs in both directions to produce XX males and XY females. Hatchery-based stock enhancement in natural waters could lead to the extermination of the wild population, depending on the sex reversal rate in the hatchery, the relative reproductive success of hatchery fish in the wild and the kind of hatchery broodstock used (wild-born or hatchery-born) [178]. Although it is generally considered that these changes in living conditions are not sufficiently drastic to lead to the extinction of wild populations, their effects on effective population size and population growth cannot be neglected [178,179,188-196]. Another case of stock enhancement concerns the rainbow trout for recreational fisheries that results in a considerable number of hatchery fish released into the wild environment. Early maturity of the male rainbow trout compared to the female is a major bottleneck in production of such commercially important fish and selection experiments to increase the proportion of females by applying a temperature treatment are ongoing [177]. Magerhans et al. [197] have reported the production of female- or male-biased progenies under high-temperature treatment (18°C) versus control temperature (12°C) in different populations of rainbow trout [197]. Thus, ESR females (i.e. XY females) could also be induced in farm hatcheries for this fish species. Introduction of hatchery fish into natural waters of other commercial important fish with TSD or GSD + TE such as carp, Nile tilapia, sockeye salmon (*Oncorhynchus nerka*), chinook salmon (*Oncorhynchus tshawytcha*), European sea bass and southern flounder [5,11,177] should also be evaluated. Moreover, there is strong evidence for the presence of naturally sex-reversed individuals due to change in temperature in grayling (*Thymallus thymallus*, Salmonidae) from a wild lake [198] and in two natural populations of Nile tilapia [2]. These findings reinforce the idea that ESR (either introduced or naturally induced) should be extensively evaluated.

Fish population stability and sustainability are realistic issues in natural waters since there is a general consensus on global warming and occurrence of temperature-induced sex reversal in fish species. In 2008, Ospina-Álvarez and Piferrer [4] performed simulations to predict sex ratio shifts with temperature increases from +1.5°C to +4°C, and reported a shift from 61.7% to 78.0% of males. Moreover, the mean temperature of natural waters is projected to increase by up to ~4°C by the end of this century according to plausible global change scenarios [199], which will certainly affect sex ratios. Field studies on turtles [200] and sea turtles [201] reported significantly skewed sex ratios with modest temperature changes of 1 to 2°C. In Atlantic silverside, observations made on eggs collected from the wild have shown that a difference of 2°C in the rearing temperature during the thermosensitive period can lead to a shift in the proportion of males from 50% to 69% [202]. The resulting decrease in the proportion of females would probably affect the population structure and the viability of sensitive stocks since the reproduction potential of many fish communities is determined by the number of females available for egg production [203]. Results from long-term field investigations of grayling in a lake of Switzerland strongly suggest that the sex ratio is correlated with the average temperature that the juvenile fish experienced during their first summer and that temperature change was involved in the decline of the population [198]. For some researchers, it is possible that sensitive species, including species with TSD, will not be able to adapt fast enough to the changes in temperature due to global warming [200,204], these changes being characterized by their fast pace [199]. Thus, global temperature fluctuations will have detrimental effects on fish populations, especially the thermosensitive species. However, an experiment in Atlantic silverside showed that TSD could rapidly evolve in response to selection because a balanced sex ratio was reached after 8 to 10 generations by increasing repeatedly the number of individuals with the minority sex in an extreme and constant temperature environment [181]. This result, although limited to one species, indicates that fish may be able to adapt rapidly to changing temperature conditions.

As discussed in this article, descriptive and comparative analyses of mRNA expression patterns have initiated research on candidate genes that participate in sex determination mechanisms, which can be classified into GSD and TSD. However, these studies are not sufficient to have a complete understanding of sex determination in vertebrates and more specifically, it is not known whether up- or down-regulation of related genes are the cause or the consequence for a female or male pathway to be followed during sex differentiation [205-212]. The hierarchical cascade and interactions of the genes that underlie GSD and TSD need to be thoroughly investigated by analyzing the expression, localization, and most important, the function of the related proteins. Overexpression and knockdown techniques allow us to investigate whether these molecular players are necessary and / or sufficient to explain the fate of sexual direction as already shown for the genes *amhr2* in fugu [50], *amhy* in Patagonian pejerrey [48], *sdY* in rainbow trout [51], and *dmy* in medaka [31,32] and the genes in other vertebrates [213-220] (Figure 2).

## Conclusions

The process of sex determination and differentiation in Teleost fish is regulated by genetic and environmental factors and their interactions. TSD is thought to occur when the water temperature experienced by the offspring irreversibly determines its primary sex. GSD occurs when primary sex is determined by the genotype at conception and is thereafter independent of environmental conditions. How and why transitions between TSD and GSD occur is still unclear but very interesting from the evolutionary point of view. So far, only a few candidate genes associated with temperature-induced sex reversal have been studied in fish species. It is assumed that the thermal master switch, which triggers the undifferentiated gonads to follow the male or female pathway, will be the gene(s) that activate the thermosensitive period or specify responses during this developmental time window. In virtue of temperature-dependent sex determination or sex differentiation, some fish species may be able to adapt rapidly to changing temperature conditions. However, certain sensitive species, including species with TSD, may not be able to adapt fast enough to the changes in temperature due to global warming. Thus, global temperature fluctuations will have detrimental effects on fish populations, especially the thermosensitive species. Descriptive and comparative analyses of gene expression patterns are not sufficient to have a complete understanding of sex determination. Hierarchical cascade and interactions of the genes that underlie GSD and TSD need to be thoroughly investigated by analyzing the expression, localization, and most important, the function of the related proteins. Overexpression and knockdown techniques allow us to examine whether these molecular players are necessary and / or sufficient to explain the fate of sexual direction. Fish possess an astonishing diversity of sex determination mechanisms contracting with systems found in mammals and birds, and they provide cheaper and better models for the broad study of mechanisms of sex determination in many cases.

## Abbreviations

TSD: Temperature-dependent sex determination; GSD + TE: Genetic sex determination plus temperature effects; TSP: Temperature sensitive period; MPT: Male-producing temperature; FPT: Female-producing temperature; MixPT: Mixed sex producing temperature; SD: Sex-determining or sex determination; DPH: Days post-hatching; amh: *Anti-Müllerian hormone* gene (also known as *mis*, *Müllerian-inhibiting substance* gene); amhr2: *Anti-Müllerian hormone receptor type II* gene; amhY: *Y-linked anti-Müllerian hormone* gene; cyp17: *Cytochrome P450, family 17* gene; cyp19: *Cytochrome P450, family 19* gene; dax1: *Dosage-sensitive sex reversal, adrenal hypoplasia critical region on the X chromosome gene 1* (also known as *Nr0b1*); dmrt1: *Doublesex and Mab 3 related transcription factor 1* gene; DMW: W-linked DM-domain; dmy: *Y-specific DM-domain gene 9* (*dmrt1Y*); fgf9: *Fibroblast growth factor 9* gene; foxl2: *Forkhead box protein L2* gene; gsdf: *Gonadal soma-derived growth factor* gene; rspo1: *R-spondin 1* gene; sdY: *Sexually dimorphic on the Y-chromosome* gene is a Y-linked, truncated, divergent form of the *interferon regulatory factor 9* gene (*irf9*); sf-1: *Steroidogenic factor 1* (also known as *ad4bp* or *nr5a1*); sox8: *SRY-like HMG-box containing transcription factor 8* gene; sox9: *SRY-like HMG-box containing transcription factor 9* gene; SRY: Sex-determining region on the Y chromosome; TGF-β: Transforming growth factor β; wnt4: *Wingless integration site family member 4* gene; wt1: *Wilms tumor 1* gene.

## Competing interests

The authors declare that they have no competing interests.

## Authors’ contributions

HPW and ZGS conceived the review. ZGS was responsible for drafting and revising the manuscript. HPW was involved in drafting the manuscript and in critical and constructive revisions. Both authors read and approved the final manuscript.

## Supplementary Material

Additional file 1 **Overview of the literature on the effects of temperature on sex ratios and related gene expression in fish.** The data provided show the temperature effects on sex ratio and expression of related genes, *dmrt1* and *cyp19a1a* during undifferentiated, differentiating, and differentiated gonads in different fish species √: expressed; ×: not expressed; NS: not studied [4,42,83,86,170,133,135-138,213],[205]. Click here for file
