# Supplementary material for: Molecular players involved in temperature-dependent sex determination and sex differentiation in Teleost fish
Source: Genet Sel Evol. 2014 Apr 15;46(1):26. doi: 10.1186/1297-9686-46-26 (PMC4108122; doi:10.1186/1297-9686-46-26)
Supplement: Additional file 1 — Overview of the literature on the effects of temperature on sex ratios and related gene expression in fish. The data provided show the temperature effects on sex ratio and expression of related genes, dmrt1 and cyp19a1a during undifferentiated, differentiating, and differentiated gonads in different fish species √: expressed; ×: not expressed; NS: not studied [4,42,83,86,170,133,135-138,213],[205]. [file 1297-9686-46-26-S1.docx]

1. Overview of the literature on the effects of temperature on sex ratios and related gene expression in fish

| **Genes**  **Species** | **Temperature**  **(℃)** | **Treatment period** | **TSP** | **Sex ratio**  **(%M-%F)** | **Onset of sex differentiation** | **Gene expression during gonadal development** | | | **Population**  **used** | **SD** | **References** |
| --- | --- | --- | --- | --- | --- | --- | --- | --- | --- | --- | --- |
|  |  |  |  |  |  | **Undifferentiated** | **Differentiating** | **Differentiated** |  |  |  |
| ***dmrt1*** |  |  |  |  |  |  |  |  |  |  |  |
| European sea bass  *Dicentrarchus labrax* | 15 FPT  20 MPT | 0-48 dph  0-66 dph | < 100 dph | 23-77  73-27 | F: 160  M: 160-250 | Not detected | ↑  ↑ | Not studied | Mixed sex | GSD+TE | [86, 170] |
| Pejerrey  *Odontesthesbonariensis* | 17 FPT  25 MixPT  29 MPT | 0-56 dph | 21-35 dph  Not studied  7-28 dph | 0-100  45-55  100-0 | 49 dph(ovary)  42 dph  42 dph (testis) | MixPT: ♂>♀,  MPT>FPT,  ♂ ↑, ♀↔ | MixPT: ♂>♀,  ♂↔, ♀, ↔  FPT ↔, MPT ↔ | Not studied | Mixed sex | TSD | [83] |
| Medaka  *Oryzias latipes* | 25  32 MPT | 5-36 Stage | 5-36 Stage | 0-100  60-40 | 36-39 Stage | Not studied | Undetected  Detected(embryos) | Not studied | AF | GSD+TE | [42] |
| ***cyp19a1a*** |  |  |  |  |  |  |  |  |  |  |  |
| Japanese flounder  *Paralichthys olivaceus* | 18 FPT  27 MPT | 30-100 dph | 30-100 dph | 0-100  100-0 | 60 dph (27-37 mm TL) | ♀=♂ | ♀»♂,  ♀↑, ♂↓ | ♀»♂ | AF | GSD+TE | [133] |
| Nile Tilapia  *Oreochromis niloticus* | 27 MixPT  35 MPT | 10-40 dpf | 12-14 dpf | AF: (2~9)-(98~91)  AM: (90~100)-(10~0)  AF:(33~86)-(67~14) | F: 28 dpf;  M: 55 dpf | ♀>♂ | 27AF>35AF>  27AM>35AM | 27AF↓, 27AM↓ | AM & AF | GSD+TE | [135] |
| Atlantic Halibut *Hippoglossus hippoglossus* | 7 MixPT  10 MixPT  13 MPT | 260–1100 ddph (15-25 mm FL) | Not studied | 51-49  58-42  62-38 | F: 38 mm FL,  M: 74 mm FL | ↑ 7>10>13  ↔ (600&1100  ↔ ddph) | Not studied | Not studied | Mixed sex | GSD+TE | [136] |
| Pejerrey  *Odontesthes bonariensis* | 17 FPT  24/25MixPT  29 MPT | 0–70 dph | 7-35 dph | 0-100  (73~27)-(27~73)  100-0 | 7-35 dph | Not studied | ↑↓↑  Bimodal  ↔ | Not studied | Mixed sex | TSD | [205] |
|  | 17 FPT  25 MixPT  29 MPT | 0-56 dph | 21-35 dph  Not studied  7-28 dph | 0-100  45-55  100-0 | 49 dph(ovary)  42 dph  42 dph (testis) | MixPT:↑,♀>♂  FPT:↑,MPT↔  MPT = FPT | MixPT: ♀>♂,  ♀↔,♂↔  FPT↓,MPT↔ | Not studied | Mixed sex | TSD | [83] |
| European seabass  *Dicentrarchus labrax* | 15 FPT  20 MPT | To 18mm TL | < 100 dph | 23-77  73-27 | F: 160  M: 160-250 | FPT = MPT  FPT↔, MPT↔ | Not studied | Not studied | Mixed sex | GSD+TE | [137] |
|  | 15 FPT  20 MPT | 3-60 dpf  10-60 dpf | 0-60 dpf | 29-71  44-56 | 150-250 dpf | Not studied | FPT = MPT,  ♀=♂ | ♀: FPT>MPT | Mixed sex | GSD+TE | [138] |

FPT: female-producing temperature; MPT: male-producing temperature; MixPT: mixed sex producing temperature; dph: days post-hatching; dpf: days post-fertilization; ddph: days degrees post-hatching; FL: fork length; TL: total length; TSP: thermosensitive period; M: male; F: female; AM: all male; AF: all female; SD: sex determination; GSD: genetic sex determination; TSD: temperature-dependent sex determination; TE: temperature effects; ↑, ↓ and ↔ denote increase, decrease and invariant expression levels, respectively. Differentiation of TSD and GSD+TE follows Ospina-Álvarez and Piferrer [4].
